# Associations of fetuin-A and osteoprotegerin with arterial stiffness and early atherosclerosis in chronic hemodialysis patients

**DOI:** 10.1186/1471-2369-14-122

**Published:** 2013-06-12

**Authors:** Panagiotis Pateinakis, Aikaterini Papagianni, Stella Douma, Georgios Efstratiadis, Dimitrios Memmos

**Affiliations:** 1 Department of Nephrology, Aristotle University of Thessaloniki, “Hippokration” General Hospital, Thessaloniki, Greece; 2 2nd Propedeutic and Department of Internal Medicine, Aristotle University of Thessaloniki, “Hippokration” General Hospital, Thessaloniki, Greece

**Keywords:** Intima-media thickness, Fetuin-A, Hemodialysis, Osteoprotegerin, Pulse wave velocity

## Abstract

**Background:**

Cardiovascular morbidity and mortality remains excessive in patients with chronic kidney disease. The association of vascular changes with regulators of extraosseous calcification in this patient population is still under investigation. The aim of the present study was to investigate the associations of the calcification inhibitor fetuin-A, and the anti-osteoclastic factor osteoprotegerin (OPG) with vascular pathology in chronic hemodialysis patients.

**Methods:**

In this cross-sectional study including 81 stable chronic hemodialysis patients, we measured carotid-to-femoral pulse wave velocity (cfPWV) with applanation tonometry, reflecting arterial stiffness, and common carotid intima-media thickness (ccIMT), a surrogate of early atherosclerosis, as well as serum levels of fetuin-A and OPG. Co-morbidities, traditional cardiovascular risk factors, inflammatory markers and mineral-bone disease serology parameters were also recorded.

**Results:**

cfPWV correlated inversely with fetuin-A (r=−0.355, p=0.001) and positively with OPG (r=0.584, p<0.001). In multilinear regression analysis including age, gender, diabetes, cardiovascular disease, hypertension, pulse pressure, LDL, logCRP, both fetuin-A and OPG were independently associated with cfPWV (p=0.024 and p=0.041 respectively). ccIMT was negatively associated with fetuin-A (r=−0.312, p=0.005) and positively with OPG (r=0.521, p<0.0001); however these associations lost statistical significance after adjustment for age.

**Conclusion:**

In chronic hemodialysis patients both fetuin-A and OPG levels are independently associated with arterial stiffness but not with early atherosclerotic vascular changes.

## Background

Cardiovascular pathology remains the leading cause of morbidity and mortality in patients with chronic kidney disease (CKD) [1]. Two types of vascular changes are observed, atherosclerotic and arteriosclerotic [2]. Atherosclerosis is associated with increase of the arterial intima–media thickness (IMT), leading eventually to luminal obstruction with consequent ischemic events, such as myocardial infarction and stroke. Arteriosclerosis leads to arterial stiffening and increased pulse wave velocity (PWV) and pulse pressure, resulting in left ventricular hypertrophy and reduced coronary perfusion. In CKD patients both atherosclerosis and arteriosclerosis appear early and follow an accelerated course contributing to the excessive cardiovascular mortality in this patient population [3,4].

The pathogenesis of vasculopathy in end-stage renal disease (ESRD) involves traditional risk factors of atherosclerosis (older age, hypertension, dyslipidemia, diabetes mellitus), which are highly prevalent in this patient population, but also several uremia-related risk factors (inflammation, oxidative stress, mineral and bone disorders), which are still under investigation [5].

Recent evidence suggests that vascular calcification is a highly regulated active process, and that the phenotypic trans-differentiation of vascular smooth muscle cells (VSMCs) into osteoblast-like cells is a key pathogenetic event [5]. A variety of factors that influence this process have been recognized, including dysregulated mineral homeostasis and bone turnover, as well as imbalance between promoters and inhibitors of extra-osseous bone formation [5]. Thus it appears that there is an interaction between bone metabolism and vascular health, placing current understanding of vascular pathophysiology on a bone-vascular axis [5].

Fetuin-A is a liver-derived potent systemic inhibitor of calcification and a negative acute phase reactant [6]. Apart from preventing calcium and phosphate precipitation in the serum, fetuin-A protects from arterial media calcification by inhibiting VSMC apoptosis and preventing basic calcium particle nucleation in the extracellular matrix [7]. In hemodialysis (HD) patients, low fetuin-A levels have been associated with severe and extensive vascular calcification, as well as with increased all-cause and cardiovascular mortality [8].

Osteoprotegerin (OPG) is a soluble member of the tumor necrosis factor (TNF) receptor superfamily, produced by osteoblasts and vascular endothelial and smooth muscle cells [9]. It has an anti-osteoclastic effect being a soluble decoy receptor for the osteoclast activator RANKL (receptor activator of nuclear factor-κB ligand). Moreover, it appears to be an important regulator of vascular calcification [10] and in HD patients, high OPG levels have been associated with vascular calcification and mortality [11].

The precise roles of fetuin-A and OPG in the development of uremic vasculopathy are not fully elucidated [12]. The aim of the present study was to investigate the associations of serum fetuin-A and OPG levels with carotid to femoral PWV (cfPWV), a marker of arteriosclerosis [13], and common carotid IMT (ccIMT), a marker of early atherosclerosis [14], in a group of maintenance HD patients.

## Methods

### Patients

Eighty one adult patients (52 male, mean age 59.8 years, range 22–86 years) on chronic maintenance HD for at least three months (mean HD duration 66 months, range 3–274 months) were included in the study. All patients were from the Dialysis Unit of the University Department of Nephrology at “Hippokration” General Hospital who consented to the protocol and entered the study consecutively. Chronic renal failure was attributed to glomerulonephritis in 21 cases (25.9%), diabetes mellitus in 17 (21%), tubulointerstitial nephritis in 10 (12.3%), polycystic kidney disease in 8 (10.0%), renovascular hypertension in 7 (8.6%), and was undetermined in 18 cases (22.2%). Patients were clinically stable without active malignancy, infection, or liver disease. None of the patients was receiving antibiotics or immunosuppressive agents and none had a history of parathyroidectomy at the time of the study. All patients were receiving conventional 3.5 to 4.5-h HD, three times weekly, with synthetic (polysulphone or helixone) dialyzers, bicarbonate dialysate and tinzaparin as standard anticoagulation. Dialysis prescription was guided by a goal of achieving a value of Kt/V ≥1.2, as calculated by the second-generation Daugirdas equation. The achieved Kt/V was 1.46 ±0.21 (range 0.92 – 2.0).

The protocol conformed to the ethical guidelines of our institution and it was approved by the Institutional Review Board of “Hippokration” General Hospital. All patients signed an informed consent prior to the study entry.

### Laboratory methods

Blood samples were drawn from a peripheral vein under fasting conditions in the morning of a midweek routine dialysis session. Serum samples were separated from clotted blood by immediate centrifugation (1500g for 10 min), aliquoted and stored at −70°C until assay. Serum levels of fetuin-A and OPG were measured by an enzyme-linked immunosorbent assay (ELISA) using commercially available standard kits (human fetuin-A and human osteoprotegerin, BioVendor GmbH Heidelberg, Germany, detection limit 0.35 ng/ml and 0.10 pmol/L respectively). Serum albumin, total cholesterol, triglycerides, HDL cholesterol, LDL cholesterol, calcium, phosphorus and alkaline phosphatase were determined by routine techniques using an automated analyser (Olympus AU560, Hamburg, Germany). Levels of albumin were time averaged for the last six months before inclusion in the study. Intact parathormone (iPTH) levels were measured by radioimmunoassay (RIA). Serum CRP levels were measured by nephelometry. The detection limit was 3.75 mg/L and in the statistical evaluation all values <3.75 mg/L were treated as 3 mg/L.

### PWV measurements

cfPWV measurements were performed with the SphygmoCor® (AtCor® Medical, Sydney, Australia), by a trained operator unaware of the patient’s clinical and laboratory parameters. The device uses a high-fidelity applanation tonometer for transdermal pulse wave recording. Each subject was examined in the supine position during a mid-week non-dialysis day and within one month from blood sampling. cfPWV measurements were done in two steps. The first step involved recording the carotid pulse wave, and the second recording the femoral pulse wave, both with simultaneous ECG, for synchronization of carotid and femoral pulse wave times. Transit time between carotid and femoral pressure waves was calculated using the foot-to-foot method. Wave “foots” were identified using the intersecting tangents algorithm. Two distances on the body surface were measured, that is, from sternal notch to the femoral location and from sternal notch to the carotid location of the respective pulse wave recording sites. On entering the data into the computer, travelled distance was calculated automatically as the difference between the two distances, that is, femoral location-sternal notch minus sternal notch-carotid location. cfPWV results were expressed in meters per second (m/s) ± SD [13]. Measurements were considered reliable if the SD was ≤ 15% of the velocity value. The mean of three such measurements was considered in the analysis. In two cases cfPWV could not be reliably measured due to atrial fibrillation and impalpable femoral pulse.

### IMT measurements

Ultrasonographic studies were performed with an Aloka® Prosound A6 instrument (Aloka®, Tokyo, Japan), using a 10 MHz high-resolution probe. Each subject was examined in the supine position in a semi-dark room during a mid-week non-dialysis day within one month from blood sampling. The common carotid artery was investigated bilaterally by the same trained operator, who was unaware of the patients’ clinical and laboratory parameters. Longitudinal 2D images of the vessel were acquired, frozen in diastole and analysed offline. ccIMT was calculated as the distance between the leading edge of the lumen–intima interface and the media–adventitia interface on the far wall of the artery [14]. Measurements were performed 0.5, 1 and 2 cm below the carotid bifurcation (six measurements, three on each side) in a plaque-free arterial segment. The average measurement of the obtained values was taken as ccIMT and it was considered in the analysis.

### Blood pressure measurements

Blood pressure was recorded with an upper arm mercury sphygmomanometer according to the recommendations of the American Heart Association [15], before the cfPWV measurement. The mean value in mmHg of three measurements of systolic arterial pressure (SAP) and diastolic arterial pressure (DAP) was entered in the analysis. Pulse pressure (PP) was calculated as the difference between SAP and DAP, and mean arterial pressure (MAP) was calculated as [SAP + (2 × DAP)] / 3. Hypertension was defined as systolic blood pressure >140mmHg and/or diastolic >90mmHg or the current use of anti-hypertensive medication.

### Statistical analysis

Data are expressed as mean ± SD, median with range, or number of patients with percentage as appropriate. The associations between fetuin-A, OPG, cfPWV, ccIMT, and demographic, clinical and laboratory parameters were assessed. Normality of distribution of continuous variables was tested by the one sample Kolmogorov-Smirnov test. Non-normally distributed variables were log-transformed before entering the analysis. The significance of differences between groups was assessed by the independent samples t-test. Associations between continuous variables were tested by Pearson’s bivariate and partial correlation analyses. Multiple linear regression analysis, with simultaneous inclusion of values into the model, was used to assess the adjusted combined influence of variables on the dependent variables (cfPWV or ccIMT). Variables with p *<* 0.1 on bivariate analyses or the independent samples t-test were entered into the multiple regression models. Despite the significant bivariate correlation of SAP and PP with cfPWV, and DP and MAP with ccIMT, only PP and MAP respectively were entered in the relevant multiple regression models, in order to avoid co-linearity. The calculations were performed using SPSS for Windows® version 13.0 statistical software (SPSS®, Chicago, IL, USA). A two-tailed p value <0.05 was considered statistically significant.

## Results

The patients’ epidemiological and clinical characteristics and laboratory parameters are summarized in Tables 1 and 2.

**Table 1:** Epidemiological and clinical characteristics of 81 HD patients

Age (years)	59.8 ± 15.7
Male	52 (64.2)
Dialysis duration (months)	66.2 ± 53.9
Diabetes mellitus	17 (21)
Hypertension	57 (70.4)
History of CVD	30 (37)
Smoking	25 (30.9)
Statins	29 (35.8)
Anti-hypertensives	57 (70,4)
Anti-HPTH treatment	46 (56.8)
ESA	73 (90,1)
Intravenous iron	73 (90,1)
BMI (kg/m^2^)	24.6 ± 4.1
SAP (mmHg)	136.1 ± 18.9
DAP (mmHg)	83.6 ± 12.1
PP (mmHg)	52.9 ± 13.8
MAP (mmHg)	100.9 ± 13.4

Values are expressed as mean±SD or numbers (%) as appropriate.

*CVD* cardiovascular disease, *DAP* diastolic arterial pressure, *ESA* erythropoiesis stimulating agent, *HPTH* hyperparathyroidism, *MAP* mean arterial pressure, *PP* pulse pressure, *SAP* systolic arterial pressure.

**Table 2:** Laboratory parameters of 81 HD patients

Kt/V	1.46 ±0.21
Hb (g/dl)	11.3 ± 1.2
Creatinine (mg/dl)	9.3 ± 2.1
Calcium (mg/dl)	8.7 ± 0.7
Phosphorus (mg/dl)	5.2 ± 1.4
Ca x P (mg^2^/dl^2^)	46.4 ± 13.1
iPTH (pg/dl)	359 ± 276
APL (IU/L)	94 ± 39.8
Albumin (g/dl)	4.0 ± 0.3
Total cholesterol (mg/dl)	150.2 ± 38.5
HDL (mg/dl)	44.9 ± 14.1
LDL (mg/dl)	73.8 ± 31.7
Triglycerides (mg/dl)	139.7 ± 63.6
CRP (mg/L)	7.2 ± 9.3
OPG (pmol/L)	18.498 ± 8.198
Fetuin-A (g/L)	0.713 ± 0.147
cfPWV (m/s)	9.91 ± 2.29
ccIMT (mm)	0.833 ± 0.166

Values are expressed as mean±SD.

*ALP* alkaline phosphatase, *cfPWV* carotid to femoral pulse wave velocity, *CRP* C-reactive protein, *ccIMT* common carotid intima–media thickness, *HDL* high density lipoprotein, *iPTH* intact parathormone, *LDL* low density lipoprotein, *OPG* osteoprotegerin.

### Correlations of fetuin-a and OPG levels

Fetuin-A correlated negatively with age (r=−0.308, p=0.005), and logCRP (r=−0.365, p=0.001), and positively with albumin (r=0.243, p=0.029) and smoking habit (p=0.039, 95%CI −0.142 to −0.004).

OPG correlated positively with age (r=0.677, p<0.001), dialysis duration (r=0.225, p=0.046), SAP (r=0.234, p=0.038) and PP (r=0.339, p=0.002), and negatively with BMI (r=−0.32, p=0.004) and albumin (r=−0.263, p=0.019).

There was a non-significant negative association between fetuin-A and OPG levels (r=−0.175, p=0.123).

### Correlations of cfPWV with clinical and laboratory parameters, fetuin-A and OPG levels

Arterial stiffness was associated with history of cardiovascular disease (CVD) (p=0.003, 95% CI=−2.57 to −0.55), hypertension (p=0.024, 95% CI=−2.37 to −0.17) and diabetes mellitus (p=0.039, 95% CI=−2.62 to −0.07), and correlated positively with age (r=0.589, p<0.001), SAP (r=0.306, p=0.006), PP (r=0.403, p<0.001), and LDL levels (r=0.242, p=0.037) (Table 3).

**Table 3:** Variables associated with cfPWV, in univariate and multiple regression analysis

**Variable**	**Univariate**	Multiple regression (**)	
	p(*)	p	standard β
Gender	0.973	0.150	−0.128
Age	<0.001	0.018	0.267
Diabetes mellitus	0.039	0.063	0.154
History of CVD	0.003	0.220	0.107
Hypertension	0.024	0.052	0.166
SAP	0.006	not entered	
PP	<0.001	0.010	0.240
LDL	0.037	0.032	0.183
*log*CRP	0.051	0.481	0.063
Fetuin-A	0.001	0.032	−0.197
OPG	<0.001	0.041	0.227

(*)Pearson’s correlation for linear association of continuous variables, and independent samples t-test for difference between groups, (**)Model adjusted R^2^=0.549, p<0.001. *cfPWV*carotid-femoral pulse wave velocity, *CRP* C-reactive protein, *CVD* cardiovascular disease, *LDL* low density lipoprotein, *OPG* osteoprotegerin, *PP* pulse pressure, *SAP* systolic arterial pressure.

**Figure 1:**
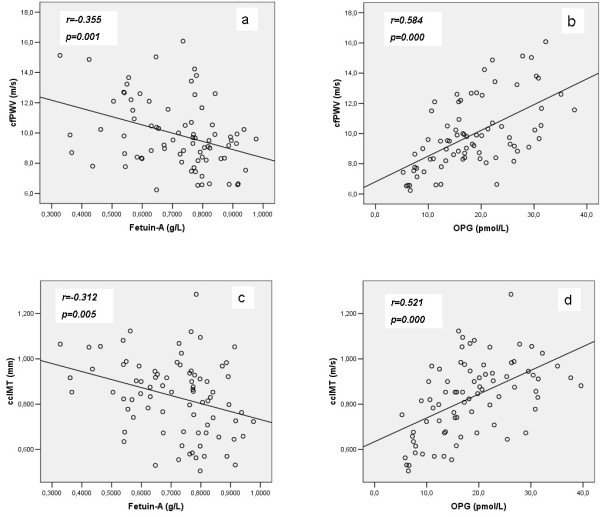
Correlations between cfPWV and ccIMT and fetuin-A (1a and 1c respectively) and OPG (1b and 1d respectively) in chronic HD patients.

Arterial stiffness also showed a significant negative correlation fetuin-A levels (r=−0.355, p=0.001) (Figure 1a), and a strong positive correlation with OPG levels (r=0.584, p<0.001) (Figure 1b). In partial correlation analysis these associations were independent of age, gender, dialysis duration, SAP, PP, LDL levels and co-morbidities, such as diabetes mellitus, hypertension and history of CVD.

**Figure 2:**
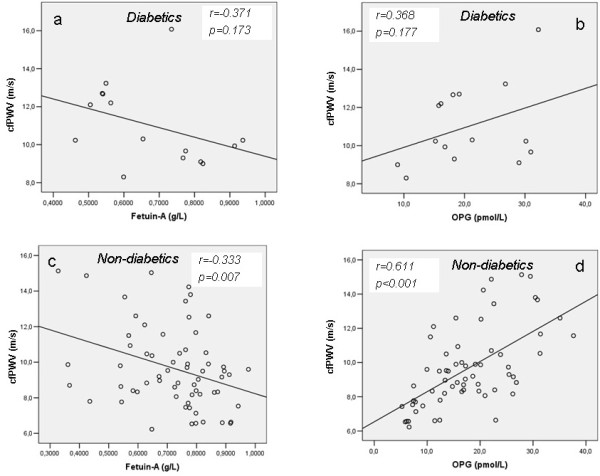
Correlations between cfPWV and fetuin-A and OPG in diabetic (2a and 2b respectively) and non-diabetic (2c and 2d respectively) chronic HD patients.

In diabetic patients (n=17, 21%) the correlations of cfPWV with both fetuin-A and OPG did not reach statistical significance (p=0.173 and p=0.177 respectively) (Figure 2a and 2b), while in non-diabetic patients (n=64, 79%) the above correlations were highly significant (p=0.007 and p<0.001 respectively) (Figure 2c and 2d).

In a multiple regression model including age, gender, diabetes mellitus, history of CVD, hypertension, PP, LDL, logCRP, both fetuin-A and OPG retained their significant association with cfPWV (p=0.032 and p=0.041 respectively), along with age, PP and LDL (Table 3).

### Correlations of ccIMT with clinical and laboratory parameters, fetuin-a and OPG levels

ccIMT was associated with the presence of diabetes mellitus (p=0.005, 95% CI=−0.21 to −0.04) and history of CVD (p=0.001, 95% CI=−0.19 to −0.05), and correlated significantly with age (r=0.744, p<0.001), DAP (r=-0.299, p=0.008), MAP (r=-0.238, p=0.032), serum albumin (r=−0.293, p=0.008) and logCRP (r=0.285, p=0.01) (Table 4).

**Table 4:** Variables associated with ccIMT, in univariate and multiple regression analysis

**Variable**	**Univariate**	Multiple regression (**)	
	p(*)	p	standard β
Gender	0.058	0.025	−0.163
Age	<0.001	<0.001	0.581
Diabetes mellitus	0.005	0.032	0.154
CVD	0.001	0.046	0.158
DAP	0.008	not entered	
MAP	0.032	0.117	−0.124
Albumin	0.008	0.357	0.075
*log*CRP	0.010	0.875	0.013
Fetuin-A	0.001	0.201	−0.103
OPG	<0.001	0.389	0.086

(*)Pearson’s correlation for linear association of continuous variables and independent samples t-test for difference between groups. (**)Model adjusted R^2^=0.615, p<0.001.

*ccIMT* common carotid intima-media thickness, *CRP* C-reactive protein, *CVD* cardiovascular disease, *DAP* diastolic arterial pressure, *MAP* mean arterial pressure, *OPG* osteoprotegerin.

ccMT also showed a significant negative correlation with fetuin-A levels (r=−0.312, p=0.005) (Figure 1c), and a strong positive correlation with OPG levels (r=0.521, p<0.001) (Figure 1d). In partial correlation analysis these associations were independent of gender, dialysis duration, DAP, MAP, diabetes mellitus, history of CVD, and logCRP, but lost significance after adjustment for age.

**Figure 3:**
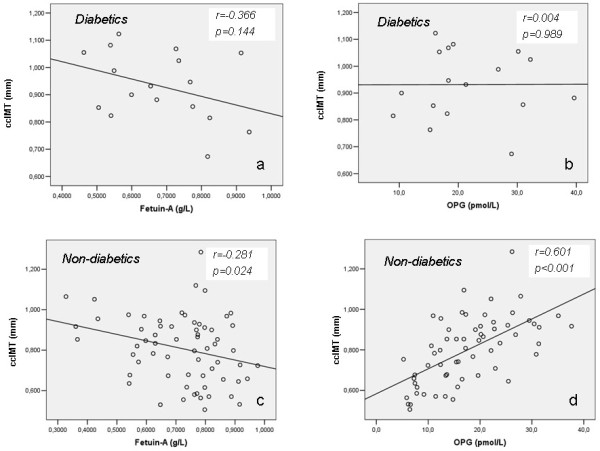
Correlations between ccIMT and fetuin-A and OPG in diabetic (3a and 3b respectively) and non-diabetic (3c and 3d respectively) chronic HD patients.

In diabetic patients (n=17, 21%) the correlations of ccIMT with fetuin-A and OPG did not reach statistical significance (p=0.144 and p=0.989 respectively) (Figure 3a and 3b) whereas in non-diabetic patients (n=64, 79%) these associations were significant (p=0.024 and p<0.001 respectively) (Figure 3c and 3d).

In multiple regression analysis only age, gender, diabetes mellitus and history of CVD were independently associated with ccIMT (Table 4). In a multiple regression model including only laboratory parameters (albumin, logCRP, fetuin-A and OPG, adjusted r^2^=0.305, p<0.001), OPG was the only variable significantly correlated with ccIMT (p<0.001, standard β=0.452).

## Discussion

Fetuin-A is a potent systemic inhibitor of calcium phosphate precipitation, that binds hydroxyapatite structures, thus protecting VSMCs from the effects of calcium overload and calcification [16]. Recent studies have shown that serum fetuin-A levels are lower in elderly HD patients compared with controls [17] and are inversely correlated with valvular calcification [18]. Moreover, lower fetuin-A levels were found to be associated with increased vascular calcification evaluated by computed tomography (CT) in young HD patients [12] and in elderly community dwellers free of prevalent CVD [19]. However, a recent study in prevalent adult HD patients [20] failed to show any correlation between fetuin-A levels and coronary artery and abdominal aortic calcification scores evaluated by CT scans.

Fetuin-A mediated prevention of VSMC calcification could potentially attenuate hemodynamic consequences of vascular calcification, such as arterial stiffening and increased PWV [16]. In the present study in chronic HD patients, serum fetuin-A levels were independently associated with cfPWV. The results of previous studies which investigated the above association are inconsistent. Mori et al. [21] found an independent positive correlation between fetuin-A and common carotid artery stiffness, in healthy Japanese subjects, while Roos et al. [22] reported an inverse association with aortic PWV in men, but not in women, with normal renal function. Regarding CKD patients, no association between cfPWV and fetuin-A was found by Schlieper et al. [23] in HD patients and by Porażko et al. [24] in peritoneal dialysis and pre-dialysis ESRD patients. Moreover, Hermans et al. [25] demonstrated, in HD patients, a significant inverse correlation, which, however, lost significance after adjustment for age, gender, MAP and diabetes mellitus. It should be noted that in the latter study, patients had fetuin-A levels comparable with controls and low levels of inflammatory activity. Finally, the studies by Schroff et al. [12] in children and by Porażko et al. [24] in adult HD patients, are in agreement with our findings. The above discrepant results may, at least partially, reflect differences in methodology and patient population. However, the independent negative association of cfPWV with fetuin-A is in accordance with its well documented anti-calcifying effects [6,7,16], and its reported negative association with mortality [8].

In the present study, fetuin-A also showed a significant negative association with ccIMT, which however, lost significance after adjustment for age. Studies investigating the above association are limited and the results are inconsistent. A recent study in patients with essential hypertension and normal renal function showed an independent negative correlation of fetuin-A with carotid IMT [26]. However, an earlier study in community-living individuals without prevalent CVD failed to demonstrate a similar association [19], while in 90 patients with carotid or femoral atherosclerosis and preserved renal function a positive correlation was observed [27]. Caglar et al. showed a negative correlation of fetuin-A with carotid IMT in non-diabetic patients with CKD stage 1–5 which, however, lost significance in multivariate analysis [28]. Another study reported that lower fetuin-A levels independently predicted higher carotid IMT in children and adolescents with ESRD, but not with CKD stages 2–4 [29]. In HD patients, one study found no correlation [23], and in another the correlation lost statistical significance after adjustment for age [30], as in the present report. However in a study by Pertosa et al. [31] baseline fetuin-A levels were inversely and independently associated with carotid IMT measurements two years later. Differences in methodology and patient population may again, at least partially, account for the discrepant results. Furthermore, in contrast to the well documented anti-calcifying effects of fetuin-A, its association with early atherosclerotic vascular changes appears to warrant further investigation. However, it should be noted that an association was reported between fetuin-A and endothelial dysfunction. Caglar et al. [32] demonstrated an independent positive correlation between fetuin-A levels and brachial artery endothelium-dependent vasodilatation (FMD), both before and after renal transplantation. In addition, another study in non-diabetic patients with CKD stage 1–5 demonstrated that endothelial dysfunction worsened in parallel to the reduction in the estimated glomerular filtration rate and it was independently associated with serum fetuin-A levels [28]. Nevertheless, in prevalent HD patients lower fetuin-A levels have been associated with increased risk for vascular complications such as loss of arteriovenous access patency [33] and stroke [34].

Fetuin-A is a negative acute phase reactant and its associations with cardiovascular morbidity and mortality might be influenced by the presence of inflammation, a well recognised risk factor for atherosclerosis and cardiovascular events [35]. In our study, fetuin-A levels were negatively correlated with CRP, while the latter was positively correlated with ccIMT, although the latter association lost significance in multivariate analysis. Furthermore, fetuin-A has been associated with insulin resistance [36] and metabolic syndrome [37], and the presence of diabetes might also influence the associations between fetuin-A and cardiovascular risk. It should be noted that a recent prospective study in community-living individuals older than 65 years and free of CVD showed that higher fetuin-A levels were associated with lower CVD risk only among persons without type 2 diabetes, whereas a trend in the opposite direction, although non significant, was observed among diabetic individuals. Moreover, among individuals without type 2 diabetes, similar effect modification was observed by obesity and insulin resistance [38]. In our study, the correlations of fetuin-A with both cfPWV and ccIMT were significant only in the non-diabetic patients although a trend in the same direction was also observed in the diabetics. However it should be noted that the number of diabetic patients was small and studies in larger patient numbers are needed to confirm these findings.

OPG is produced by osteoblasts as well as by endothelial cells and VSMCs [9]. Its anti-osteoclastic properties argue for a potential role in bone-vascular crosstalk affecting vascular calcification [16] although the exact underlying mechanisms remain largely unknown. OPG concentrations in serum from CKD patients were found to be independently associated with the serum potential to induce calcification of smooth muscle cells in vitro [39]. Moreover, serum OPG levels have been independently associated with cervical artery calcification in non-dialysis CKD patients [9] and with moderate coronary artery calcification in patients with type 2 diabetes, with and without diabetic nephropathy [40]. Studies in HD patients demonstrated higher serum OPG levels compared with control subjects [17] and a positive association of OPG with arterial medial calcification assessed by plain film radiography of the pelvis [18], with coronary artery calcification score assessed by CT scan both at baseline and after 1 year [41], as well as with mortality [11]. On the contrary, results from animal studies suggested a protective role of OPG on the vasculature. Thus, OPG knockout mice develop vascular calcification which can be prevented by injection of transgenic OPG [42]. Moreover, OPG administration in rats was found to inhibit artery calcification induced by warfarin and by vitamin D [43]. The above experimental studies indicate that the positive association of OPG with vascular calcification in humans may not be necessarily causative, and it remains unclear whether increased OPG levels promote arteriosclerosis, or merely represent a compensatory mechanism aiming to attenuate further vascular injury [16,44].

In the present study an independent association was observed between cfPWV and OPG. There are few relevant published studies in dialysis patients and their results appear again inconsistent. A previous study in adult HD patients failed to document any association [23], and in another, in children on HD, the association was confounded by age [12]. However, two other studies, in accordance with our findings, demonstrated an independent association between cfPWV and OPG [10,45].

We also documented a positive association of ccIMT with OPG, even though it was not independent of age, which appeared, as expected, the strongest predictor of ccIMT. Previous studies have shown an independent positive association between OPG and carotid IMT in non-renal patients, such as healthy post-menopausal women [46], women with previous gestational diabetes [47], subjects older than 55 [48], and males with type 2 diabetes [49], but not patients with acute or chronic coronary artery disease [50]. In a recent study Janda et al. demonstrated an independent positive association of OPG with carotid IMT in 61 peritoneal dialysis patients [51]. To the best of our knowledge, no significant correlation of OPG with ccIMT has been documented in HD patients. In the present study, using a multiple regression model that included only laboratory parameters associated with ccIMT (albumin, CRP, fetuin-A and OPG), OPG was the only variable that was independently associated with ccIMT. This finding, which is in accordance with the previously reported correlation between OPG and vascular calcification and mortality, provides additional support on its association with early atherosclerosis, and might indicate a clinically relevant effect of OPG on endothelial integrity. Finally, it should be noted that in our study, OPG levels were significantly associated with both cfPWV and ccIMT only in non-diabetic patients. However, given the small number of diabetics further studies are needed to investigate the above associations in this patient population.

There are some limitations of the present study that should be considered. It is cross-sectional, which precludes validation of causative associations, and from a single centre, which affects generalizability of the results.

## Conclusion

Although in the present study, a clear distinction between arterial stiffness and atherosclerosis cannot be made, our results suggest that in stable chronic hemodialysis patients serum levels of the calcification inhibitor fetuin-A and the osteoclast inhibitor osteoprotegerin are independently associated with pulse wave velocity, a marker of arterial stiffness but not with carotid intima-media thickness, a marker of early atherosclerosis.

## Abbreviations

ccIMT: Common carotid intima-media thickness; cfPWV: Carotid to femoral pulse wave velocity; CKD: Chronic kidney disease; CRP: C-reactive protein; CT: Computed tomography; CVD: Cardiovascular disease; DAP: Diastolic arterial pressure; ELISA: Enzyme linked immunosorbent assay; FMD: Flow mediated dilatation; HD: Hemodialysis; iPTH: Intact parathormone; MAP: Mean arterial pressure; OPG: Osteoprotegerin; PP: Pulse pressure; RANKL: Receptor activator of nuclear factor κΒ; RIA: Radioimmunoassay; SAP: Systolic arterial pressure; VSMC: Vascular smooth muscle cells.

## Competing interests

The authors declare that they have no competing interests

## Authors’ contributions

PP was responsible for recruiting, data acquisition, vascular measurements, statistical analysis and drafting of the manuscript, AP conceived and supervised the study, the execution of the immunoassays, and reviewed/edited the manuscript. SD contributed to the study design and to the discussion GE contributed to the discussion and reviewed/edited the manuscript. DM supervised the study, contributed to the discussion and reviewed/edited the manuscript. All authors have read and approved the final manuscript

## Pre-publication history

The pre-publication history for this paper can be accessed here:

http://www.biomedcentral.com/1471-2369/14/122/prepub
